# Correction: Accuracy of novel antigen rapid diagnostics for SARS-CoV-2: A living systematic review and meta-analysis

**DOI:** 10.1371/journal.pmed.1003825

**Published:** 2021-10-13

**Authors:** The PLOS Medicine Staff

The third bullet under the “What did the researchers do and find?” section of the Author Summary is incorrect. The publisher apologizes for this error.

The correct text is: Across all meta-analyzed studies, when Ag-RDTs were performed according to manufacturers’ recommendations, they showed a sensitivity of 76.3% (95% CI 73.1% to 79.2%), with LumiraDx (sensitivity 88.2% [95% CI 59.0% to 97.5%]) and, of the instrument-free Ag-RDTs, Standard Q nasal (80.2% sensitivity [95% CI 70.3% to 87.4%]) performing best.
