# Supplementary figures and images for: Accuracy of novel antigen rapid diagnostics for SARS-CoV-2: A living systematic review and meta-analysis
Source: PLoS Med. 2021 Aug 12;18(8):e1003735. doi: 10.1371/journal.pmed.1003735 (PMC8389849; doi:10.1371/journal.pmed.1003735)

S1 Fig. Detailed results of the QUADAS assessment.

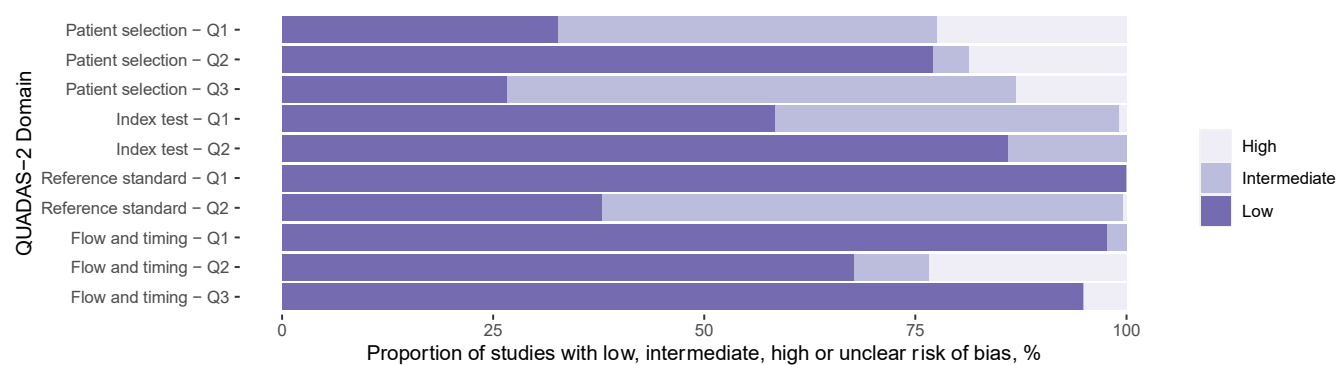

Supplement: S1 Fig — (PDF) [file pmed.1003735.s001.pdf]

S10 Fig. Funnel plot test for all data sets included in the meta-analysis.

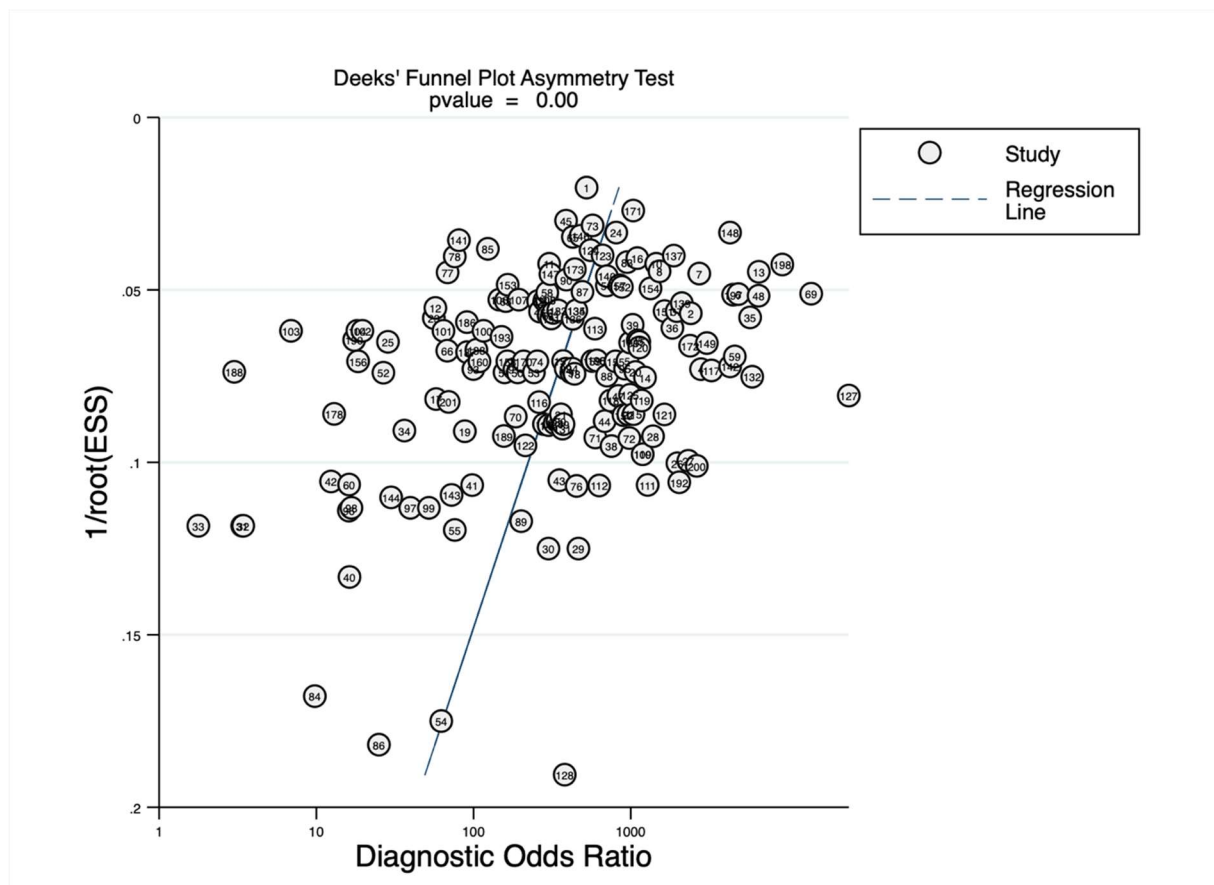

Supplement: S10 Fig — (PDF) [file pmed.1003735.s010.pdf]
