# Supplementary material for: Accuracy of novel antigen rapid diagnostics for SARS-CoV-2: A living systematic review and meta-analysis
Source: PLoS Med. 2021 Aug 12;18(8):e1003735. doi: 10.1371/journal.pmed.1003735 (PMC8389849; doi:10.1371/journal.pmed.1003735)

# S2 Fig. HSROC curve Standard Q Ag-RDT.

*Caption: HSROC = Hierarchical summary receiver-operating characteristic*

Figure A - HSROC curve Standard Q Ag-RDT

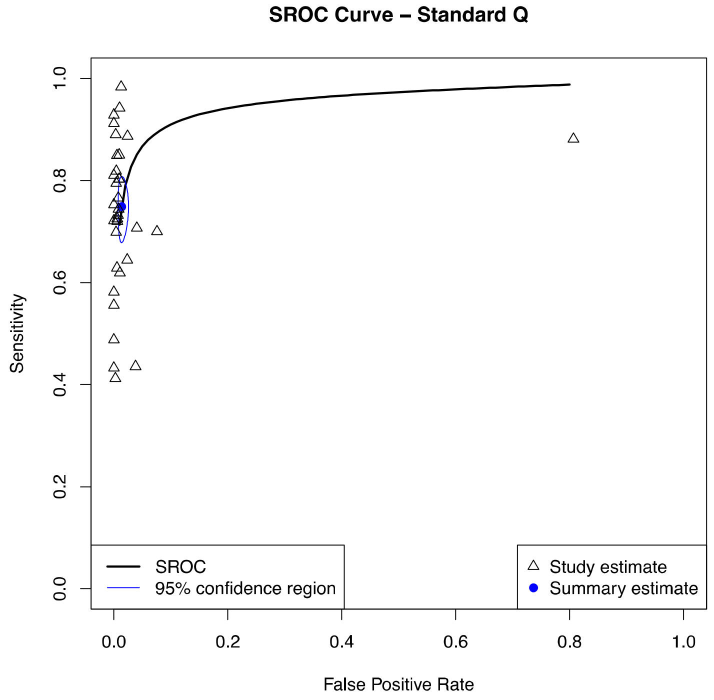


Figure B - HSROC curve LumiraDx Ag-RDT

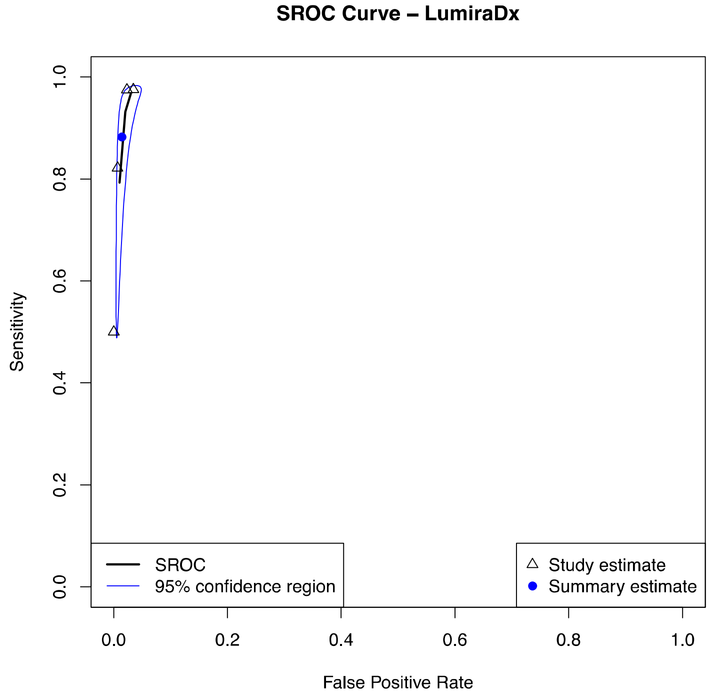

Supplement: S2 Fig — (PDF) [file pmed.1003735.s002.pdf]
