# Supplementary material for: Accuracy of novel antigen rapid diagnostics for SARS-CoV-2: A living systematic review and meta-analysis
Source: PLoS Med. 2021 Aug 12;18(8):e1003735. doi: 10.1371/journal.pmed.1003735 (PMC8389849; doi:10.1371/journal.pmed.1003735)

<u>S3 Fig. Forest plots of all Ag-RDTs.</u>

Caption: TP = true positive; FP = false positive; FN = false negative; TN = true negative; CI = confidence interval

BD Veritor

Author, Study ID	Sample size	TP	FN	TN	FP	Sensitivity [95%CI]	Specificity [95%CI]
Pekosz, a28.1	251	27	1	220	3	0.96 [0.82, 1.00]	0.99 [0.96, 1.00]
Young, a43.1	251	29	9	212	1	0.76 [0.60, 0.89]	1.00 [0.97, 1.00]
Caruana, f34.4	532	47	67	417	1	0.41 [0.32, 0.51]	1.00 [0.99, 1.00]
Stohr, f45.1	1565	86	90	1387	2	0.49 [0.41, 0.56]	1.00 [1.00, 1.00]
Schuit, f64.1	2678	149	84	2436	9	0.64 [0.57, 0.70]	1.00 [0.99, 1.00]
Kilic, f71.1	1384	77	39	1253	15	0.66 [0.57, 0.75]	0.99 [0.98, 0.99]

BinaxNOW

Author, Study ID	Sample size	TP	FN	TN	FP	Sensitivity [95%CI]	Specificity [95%CI]
Pilarowski, a29.1	878	15	11	852	0	0.58 [0.37, 0.77]	1.00 [1.00, 1.00]
Pollock, f17.1	2308	226	66	2004	12	0.77 [0.72, 0.82]	0.99 [0.99, 1.00]
James, f23.1	2339	86	66	2184	3	0.57 [0.48, 0.65]	1.00 [1.00, 1.00]
Okoye, f51.1	2638	24	21	2593	0	0.53 [0.38, 0.68]	1.00 [1.00, 1.00]

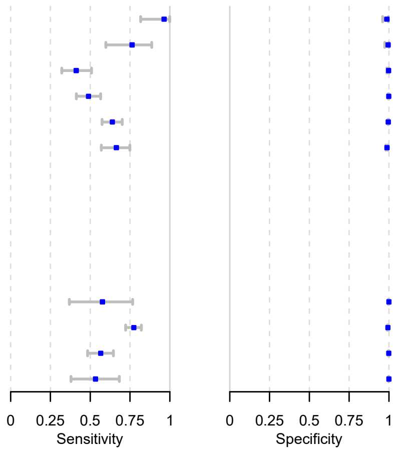

Bioeasy

Author, Study ID	Sample size	TP	FN	TN	FP	Sensitivity [95%CI]	Specificity [95%CI]
Porte, a31.1	127	77	5	45	0	0.94 [0.86, 0.98]	1.00 [0.92, 1.00]
Weitzel, a41.4	111	68	12	31	0	0.85 [0.75, 0.92]	1.00 [0.89, 1.00]
Parada−Ricart, a58.1	172	19	7	125	21	0.73 [0.52, 0.88]	0.86 [0.79, 0.91]

Rapigen

Author, Study ID	Sample size	TP	FN	TN	FP	Sensitivity [95%CI]	Specificity [95%CI]
Schildgen, a33.1	73	14	28	27	4	0.33 [0.20, 0.50]	0.87 [0.70, 0.96]
Shrestha, a36.1	113	40	7	66	0	0.85 [0.72, 0.94]	1.00 [0.95, 1.00]
Weitzel, a41.1	109	49	30	30	0	0.62 [0.50, 0.73]	1.00 [0.88, 1.00]
FINDdx, a62.1	476	87	30	355	4	0.74 [0.66, 0.82]	0.99 [0.97, 1.00]
Shidlovskaya, f61.1	106	44	34	28	0	0.56 [0.45, 0.68]	1.00 [0.88, 1.00]
FINDdx, a62.2	1239	13	12	1214	0	0.52 [0.31, 0.72]	1.00 [1.00, 1.00]

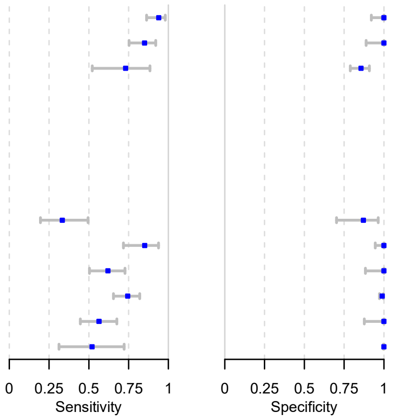

Biotical

Author, Study ID	Sample size	TP	FN	TN	FP	Sensitivity [95%CI]	Specificity [95%CI]
Favresse, f31.1	188	64	32	91	1	0.67 [0.56, 0.76]	0.99 [0.94, 1.00]

Healgen

Author, Study ID	Sample size	TP	FN	TN	FP	Sensitivity [95%CI]	Specificity [95%CI]
Favresse, f31.3	188	74	22	89	3	0.77 [0.67, 0.85]	0.97 [0.91, 0.99]

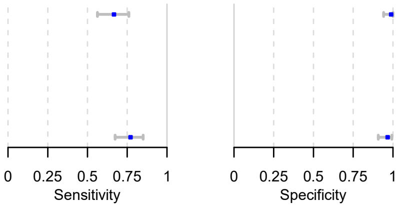

CareStart

Author, Study ID	Sample size	TP	FN	TN	FP	Sensitivity [95%CI]	Specificity [95%CI]
Pollock, f59.1	1498	135	99	1243	21	0.58 [0.51, 0.64]	0.98 [0.98, 0.99]

SGTI−flex

Author, Study ID	Sample size	TP	FN	TN	FP	Sensitivity [95%CI]	Specificity [95%CI]
Shidlovskaya, f61.2	106	41	37	27	1	0.53 [0.41, 0.64]	0.96 [0.82, 1.00]

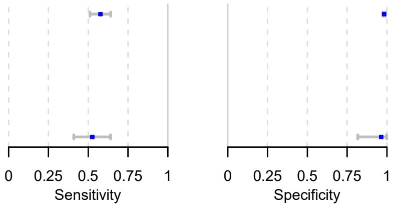

CLINITEST

Author, Study ID	Sample size	TP	FN	TN	FP	Sensitivity [95%CI]	Specificity [95%CI]
Olearo, a54.4	184	46	38	100	0	0.55 [0.44, 0.66]	1.00 [0.96, 1.00]
Torres, f29.1	178	73	18	87	0	0.80 [0.71, 0.88]	1.00 [0.96, 1.00]
Torres, f29.2	92	15	10	67	0	0.60 [0.39, 0.79]	1.00 [0.95, 1.00]
Baro, f33.2	286	52	49	182	3	0.52 [0.41, 0.62]	0.98 [0.95, 1.00]

R−Biopharm

Author, Study ID	Sample size	TP	FN	TN	FP	Sensitivity [95%CI]	Specificity [95%CI]
Toptan, a55.1	67	45	13	9	0	0.78 [0.65, 0.88]	1.00 [0.66, 1.00]
Toptan, a55.2	70	16	16	38	0	0.50 [0.32, 0.68]	1.00 [0.91, 1.00]
Kohmer, f32.1	100	29	45	25	1	0.39 [0.28, 0.51]	0.96 [0.80, 1.00]

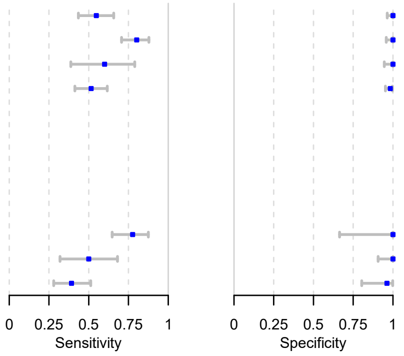

E25 Bio

Author, Study ID	Sample size	TP	FN	TN	FP	Sensitivity [95%CI]	Specificity [95%CI]
Pickering, f73.2	200	75	25	86	14	0.75 [0.65, 0.83]	0.86 [0.78, 0.92]

Spring

Author, Study ID	Sample size	TP	FN	TN	FP	Sensitivity [95%CI]	Specificity [95%CI]
Pickering, f73.4	200	77	23	98	2	0.77 [0.68, 0.85]	0.98 [0.93, 1.00]

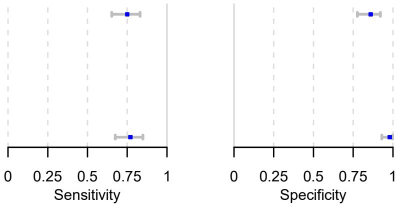

Ecotest

Author, Study ID	Sample size	TP	FN	TN	FP	Sensitivity [95%CI]	Specificity [95%CI]
Homza, f87.1	318	81	26	204	7	0.76 [0.66, 0.84]	0.97 [0.93, 0.99]

ND COVID

Author, Study ID	Sample size	TP	FN	TN	FP	Sensitivity [95%CI]	Specificity [95%CI]
Homza, f87.3	191	54	23	64	50	0.70 [0.59, 0.80]	0.56 [0.47, 0.65]

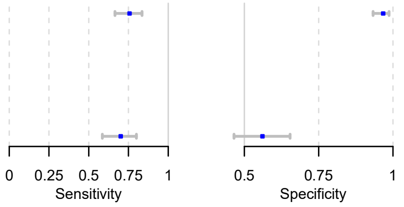

Elecsys

Author, Study ID	Sample size	TP	FN	TN	FP	Sensitivity [95%CI]	Specificity [95%CI]
Nörz, f78.1	3139	236	156	2743	4	0.60 [0.55, 0.65]	1.00 [1.00, 1.00]

Dräger

Author, Study ID	Sample size	TP	FN	TN	FP	Sensitivity [95%CI]	Specificity [95%CI]
Osmanodja, f79.1	379	62	8	308	1	0.89 [0.79, 0.95]	1.00 [0.98, 1.00]

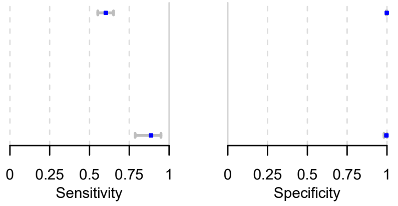

Encode

Author, Study ID	Sample size	TP	FN	TN	FP	Sensitivity [95%CI]	Specificity [95%CI]
Pickering, f73.5	200	74	26	100	0	0.74 [0.64, 0.82]	1.00 [0.96, 1.00]

SureScreen F

Author, Study ID	Sample size	TP	FN	TN	FP	Sensitivity [95%CI]	Specificity [95%CI]
Pickering, f73.6	200	69	31	98	2	0.69 [0.59, 0.78]	0.98 [0.93, 1.00]

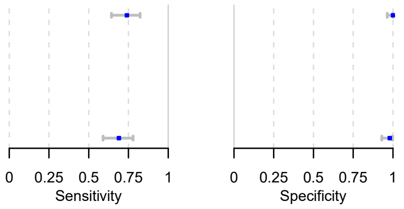

Espline

Author, Study ID	Sample size	TP	FN	TN	FP	Sensitivity [95%CI]	Specificity [95%CI]
Takeda, a50.1	162	50	12	100	0	0.81 [0.69, 0.90]	1.00 [0.96, 1.00]
Sberna, f83.1	136	5	57	74	0	0.08 [0.03, 0.18]	1.00 [0.95, 1.00]
FINDdx, f92.1	723	88	24	611	0	0.79 [0.70, 0.86]	1.00 [0.99, 1.00]

MEDsan

Author, Study ID	Sample size	TP	FN	TN	FP	Sensitivity [95%CI]	Specificity [95%CI]
Olearo, a54.3	184	38	46	97	3	0.45 [0.34, 0.56]	0.97 [0.92, 0.99]
Wagenhäuser, f89.3	3221	23	40	3146	12	0.36 [0.25, 0.50]	1.00 [0.99, 1.00]

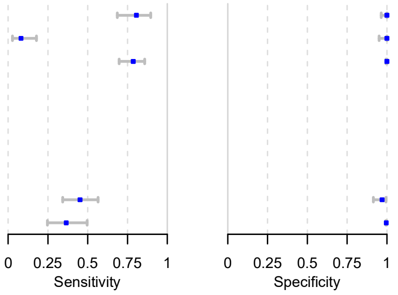

Exdia

Author, Study ID	Sample size	TP	FN	TN	FP	Sensitivity [95%CI]	Specificity [95%CI]
Caruana, f34.3	532	55	59	416	2	0.48 [0.39, 0.58]	1.00 [0.98, 1.00]

iChroma

Author, Study ID	Sample size	TP	FN	TN	FP	Sensitivity [95%CI]	Specificity [95%CI]
FINDdx, f39.1	232	30	11	191	0	0.73 [0.57, 0.86]	1.00 [0.98, 1.00]

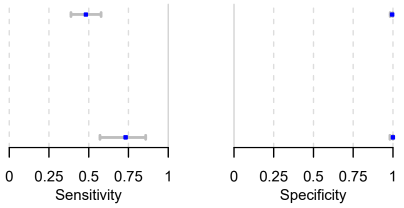

Joysbio

Author, Study ID	Sample size	TP	FN	TN	FP	Sensitivity [95%CI]	Specificity [95%CI]
FINDdx, f40.1	265	31	13	219	2	0.70 [0.55, 0.83]	0.99 [0.97, 1.00]
Homza, f87.2	225	52	38	133	2	0.58 [0.47, 0.68]	0.98 [0.95, 1.00]

Wondfo

Author, Study ID	Sample size	TP	FN	TN	FP	Sensitivity [95%CI]	Specificity [95%CI]
FINDdx, f41.1	328	48	8	272	0	0.86 [0.74, 0.94]	1.00 [0.99, 1.00]

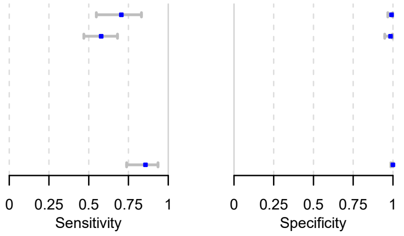

Lepu

Author, Study ID	Sample size	TP	FN	TN	FP	Sensitivity [95%CI]	Specificity [95%CI]
Baro, f33.4	286	46	55	165	20	0.46 [0.36, 0.56]	0.89 [0.84, 0.93]

SureScreen V

Author, Study ID	Sample size	TP	FN	TN	FP	Sensitivity [95%CI]	Specificity [95%CI]
Baro, f33.5	286	29	72	181	4	0.29 [0.20, 0.39]	0.98 [0.95, 0.99]
Pickering, f73.3	200	65	35	100	0	0.65 [0.55, 0.74]	1.00 [0.96, 1.00]

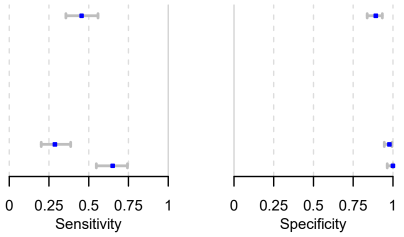

Nadal

Author, Study ID	Sample size	TP	FN	TN	FP	Sensitivity [95%CI]	Specificity [95%CI]
Kohmer, f32.3	100	18	56	26	0	0.24 [0.15, 0.36]	1.00 [0.87, 1.00]
Wagenhäuser, f89.1	806	13	10	783	0	0.56 [0.34, 0.77]	1.00 [1.00, 1.00]
FINDdx, f94.1	462	61	8	390	3	0.88 [0.78, 0.95]	0.99 [0.98, 1.00]

LumiraDx

Author, Study ID	Sample size	TP	FN	TN	FP	Sensitivity [95%CI]	Specificity [95%CI]
Kohmer, f32.4	100	37	37	26	0	0.50 [0.38, 0.62]	1.00 [0.87, 1.00]
Drain, f43.1	257	81	2	168	6	0.98 [0.92, 1.00]	0.97 [0.93, 0.99]
Drain, f43.2	255	39	1	210	5	0.98 [0.87, 1.00]	0.98 [0.95, 0.99]
Krüger, f58.1	761	120	26	611	4	0.82 [0.75, 0.88]	0.99 [0.98, 1.00]

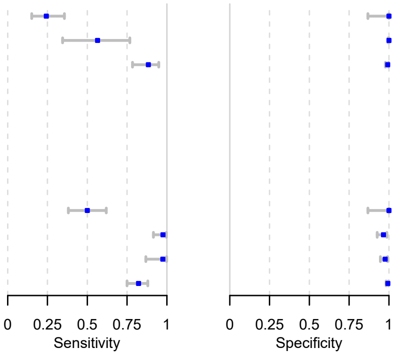

NowCheck nasal

Author, Study ID	Sample size	TP	FN	TN	FP	Sensitivity [95%CI]	Specificity [95%CI]
FINDdx, f91.1	218	71	8	137	2	0.90 [0.81, 0.96]	0.99 [0.95, 1.00]

Mologic

Author, Study ID	Sample size	TP	FN	TN	FP	Sensitivity [95%CI]	Specificity [95%CI]
FINDdx, f93.1	665	176	18	471	0	0.91 [0.86, 0.94]	1.00 [0.99, 1.00]

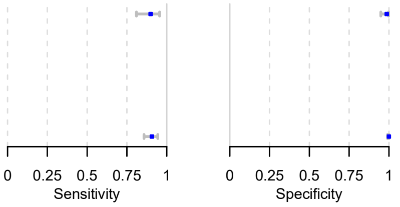

NowCheck

Author, Study ID	Sample size	TP	FN	TN	FP	Sensitivity [95%CI]	Specificity [95%CI]
FINDdx, a61.1	400	91	11	290	8	0.89 [0.81, 0.94]	0.97 [0.95, 0.99]
FINDdx, f91.2	218	71	8	137	2	0.90 [0.81, 0.96]	0.99 [0.95, 1.00]

Lumipulse G

Author, Study ID	Sample size	TP	FN	TN	FP	Sensitivity [95%CI]	Specificity [95%CI]
Basso, f10.1	234	71	16	138	9	0.82 [0.72, 0.89]	0.94 [0.89, 0.97]
Hirotsu, f47.1	1029	37	3	989	0	0.92 [0.80, 0.98]	1.00 [1.00, 1.00]
Gili, f57.1	226	86	9	120	11	0.90 [0.83, 0.96]	0.92 [0.86, 0.96]
Gili, f57.2	1738	90	0	1518	130	1.00 [0.96, 1.00]	0.92 [0.91, 0.93]
Asai, f74.1	305	49	14	238	4	0.78 [0.66, 0.87]	0.98 [0.96, 1.00]

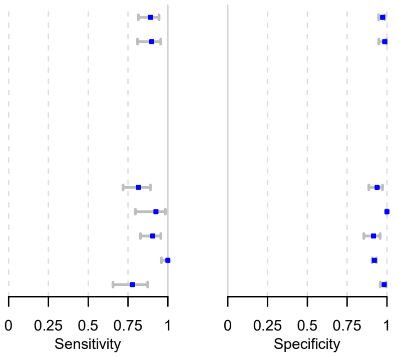

Panbio (nasal)

Author, Study ID	Sample size	TP	FN	TN	FP	Sensitivity [95%CI]	Specificity [95%CI]
FINDdx, f42.1	281	38	6	235	2	0.86 [0.73, 0.95]	0.99 [0.97, 1.00]

CerTest

Author, Study ID	Sample size	TP	FN	TN	FP	Sensitivity [95%CI]	Specificity [95%CI]
Pérez−García, f52.1	320	91	79	150	0	0.54 [0.46, 0.61]	1.00 [0.98, 1.00]

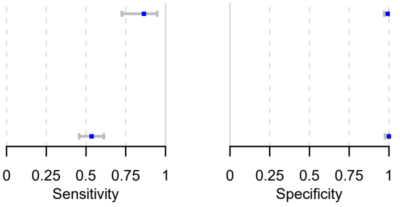

Panbio

Author, Study ID	Sample size	TP	FN	TN	FP	Sensitivity [95%CI]	Specificity [95%CI]
Abdulrahman, a01.1	4183	602	131	3420	30	0.82 [0.79, 0.85]	0.99 [0.99, 0.99]
Alemany, a02.1	919	779	55	85	0	0.93 [0.92, 0.95]	1.00 [0.96, 1.00]
Alemany, a02.2	487	93	24	365	5	0.80 [0.71, 0.86]	0.99 [0.97, 1.00]
Albert, a03.1	412	43	11	358	0	0.80 [0.66, 0.89]	1.00 [0.99, 1.00]
Berger, a05.1	535	106	18	411	0	0.86 [0.78, 0.91]	1.00 [0.99, 1.00]
Bulilete, a07.1	1362	100	40	1220	2	0.71 [0.63, 0.79]	1.00 [0.99, 1.00]
Drevinek, a10.1	591	148	75	368	0	0.66 [0.60, 0.72]	1.00 [0.99, 1.00]
Fenollar. F., a11.1	341	154	50	130	7	0.76 [0.69, 0.81]	0.95 [0.90, 0.98]
Gremmels, a12.1	1367	101	38	1228	0	0.73 [0.64, 0.80]	1.00 [1.00, 1.00]
Gremmels, a12.2	208	51	12	145	0	0.81 [0.69, 0.90]	1.00 [0.98, 1.00]
Linares, a20.1	255	44	16	195	0	0.73 [0.60, 0.84]	1.00 [0.98, 1.00]
Merino−Amador, a25.1	958	325	34	592	7	0.90 [0.87, 0.93]	0.99 [0.98, 1.00]
Schildgen, a33.2	73	21	21	24	7	0.50 [0.34, 0.66]	0.77 [0.59, 0.90]
Schwob, a35.2	271	105	17	149	0	0.86 [0.79, 0.92]	1.00 [0.98, 1.00]
Torres, a37.1	634	38	41	555	0	0.48 [0.37, 0.60]	1.00 [0.99, 1.00]
Krüger, a52.1	1034	91	13	929	1	0.88 [0.80, 0.93]	1.00 [0.99, 1.00]
Olearo, a54.2	184	37	47	100	0	0.44 [0.33, 0.55]	1.00 [0.96, 1.00]
Agullo, a56.1	652	76	56	519	1	0.58 [0.49, 0.66]	1.00 [0.99, 1.00]
Agullo, a56.2	659	59	73	527	0	0.45 [0.36, 0.54]	1.00 [0.99, 1.00]
Agullo, a56.3	610	28	93	489	0	0.23 [0.16, 0.32]	1.00 [0.99, 1.00]
Halfon, f18.1	200	72	28	99	1	0.72 [0.62, 0.80]	0.99 [0.95, 1.00]
Ngo Nsoga, f28.1	402	136	32	232	2	0.81 [0.74, 0.87]	0.99 [0.97, 1.00]
Akingba, f30.1	657	101	44	509	3	0.70 [0.62, 0.77]	0.99 [0.98, 1.00]
Favresse, f31.2	188	65	31	92	0	0.68 [0.57, 0.77]	1.00 [0.96, 1.00]
Baro, f33.1	286	39	62	184	1	0.39 [0.29, 0.49]	1.00 [0.97, 1.00]
Caruana, f34.2	532	47	67	416	2	0.41 [0.32, 0.51]	1.00 [0.98, 1.00]
FINDdx, f42.2	281	40	4	235	2	0.91 [0.78, 0.98]	0.99 [0.97, 1.00]
Domínguez Fernández, f49.1	30	19	1	10	0	0.95 [0.75, 1.00]	1.00 [0.69, 1.00]
Jääskeläinen, f50.3	190	126	26	38	0	0.83 [0.76, 0.88]	1.00 [0.91, 1.00]
Pérez−García, f52.2	320	102	68	150	0	0.60 [0.52, 0.67]	1.00 [0.98, 1.00]
Villaverde, f55.1	1620	35	42	1540	3	0.46 [0.34, 0.57]	1.00 [0.99, 1.00]
Faíco−Filho, f63.1	127	59	11	56	1	0.84 [0.74, 0.92]	0.98 [0.91, 1.00]
Stokes, f65.1	1641	231	37	1371	2	0.86 [0.81, 0.90]	1.00 [1.00, 1.00]
Del Vecchio, f66.1	1441	42	19	1379	1	0.69 [0.56, 0.80]	1.00 [1.00, 1.00]
L...Huillier, f72.1	822	78	41	702	1	0.66 [0.56, 0.74]	1.00 [0.99, 1.00]

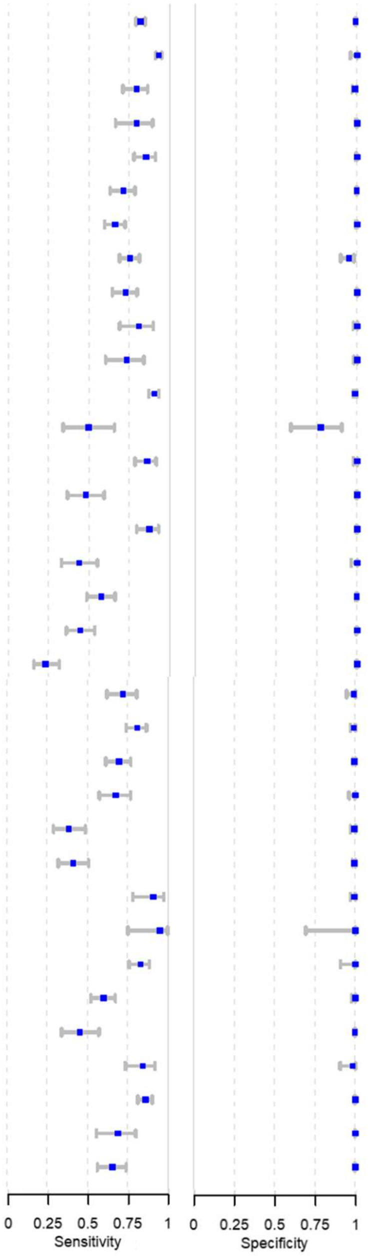

QuickNavi

Author, Study ID	Sample size	TP	FN	TN	FP	Sensitivity [95%CI]	Specificity [95%CI]
Takeuchi, f12.1	1186	91	14	1081	0	0.87 [0.79, 0.92]	1.00 [1.00, 1.00]
Takeuchi, f60.1	862	37	14	811	0	0.72 [0.58, 0.84]	1.00 [1.00, 1.00]

ECODiagnostica

Author, Study ID	Sample size	TP	FN	TN	FP	Sensitivity [95%CI]	Specificity [95%CI]
Filgueiras, f14.1	139	38	17	83	1	0.69 [0.55, 0.81]	0.99 [0.94, 1.00]

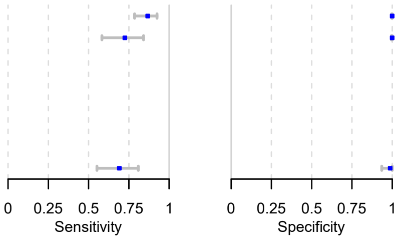

Roche

Author, Study ID	Sample size	TP	FN	TN	FP	Sensitivity [95%CI]	Specificity [95%CI]
Favresse, f31.4	188	67	29	92	0	0.70 [0.60, 0.79]	1.00 [0.96, 1.00]

VITROS

Author, Study ID	Sample size	TP	FN	TN	FP	Sensitivity [95%CI]	Specificity [95%CI]
Favresse, f31.5	188	80	16	92	0	0.83 [0.74, 0.90]	1.00 [0.96, 1.00]

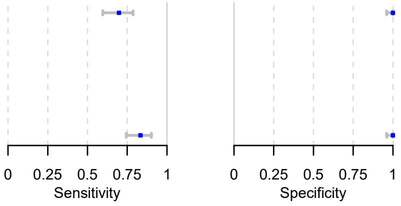

Sienna

Author, Study ID	Sample size	TP	FN	TN	FP	Sensitivity [95%CI]	Specificity [95%CI]
Bouassa, f67.1	150	90	10	50	0	0.90 [0.82, 0.95]	1.00 [0.93, 1.00]

Liaison

Author, Study ID	Sample size	TP	FN	TN	FP	Sensitivity [95%CI]	Specificity [95%CI]
Lefever, f70.1	414	138	66	210	0	0.68 [0.61, 0.74]	1.00 [0.98, 1.00]

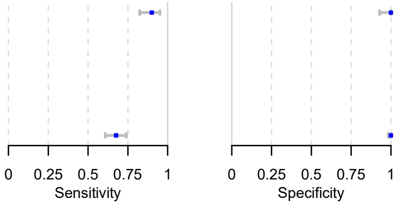

Sofia

Author, Study ID	Sample size	TP	FN	TN	FP	Sensitivity [95%CI]	Specificity [95%CI]
Beck, a04.1	346	47	14	284	1	0.77 [0.64, 0.87]	1.00 [0.98, 1.00]
Porte, a32.1	64	30	2	31	1	0.94 [0.79, 0.99]	0.97 [0.84, 1.00]
Herrera, a46.1	1172	352	107	707	6	0.77 [0.72, 0.80]	0.99 [0.98, 1.00]
Marti, f46.1	427	31	12	383	1	0.72 [0.56, 0.85]	1.00 [0.99, 1.00]
Jääskeläinen, f50.1	188	119	29	40	0	0.80 [0.73, 0.86]	1.00 [0.91, 1.00]

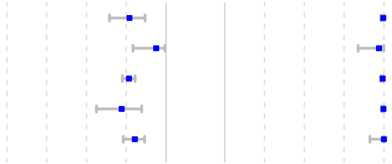

Standard Q

Author, Study ID	Sample size	TP	FN	TN	FP	Sensitivity [95%CI]	Specificity [95%CI]
Berger, a05.2	529	170	21	337	1	0.89 [0.84, 0.93]	1.00 [0.98, 1.00]
Cerutti, a08.1	185	75	29	81	0	0.72 [0.62, 0.80]	1.00 [0.96, 1.00]
Gupta, a13.1	330	63	14	252	1	0.82 [0.71, 0.90]	1.00 [0.98, 1.00]
Igl...i, a15.1	970	158	28	780	4	0.85 [0.79, 0.90]	1.00 [0.99, 1.00]
Krüttgen, a16.1	150	53	22	72	3	0.71 [0.59, 0.81]	0.96 [0.89, 0.99]
Krüger, a17.1	1263	36	11	1207	9	0.77 [0.62, 0.88]	0.99 [0.99, 1.00]
Lindner, a21.1	289	29	10	248	2	0.74 [0.58, 0.87]	0.99 [0.97, 1.00]
Lindner, a21.2	289	31	8	249	1	0.80 [0.64, 0.91]	1.00 [0.98, 1.00]
Nalumansi, a27.1	262	63	27	159	13	0.70 [0.59, 0.79]	0.92 [0.87, 0.96]
Schildgen, a33.3	73	37	5	6	25	0.88 [0.74, 0.96]	0.19 [0.07, 0.38]
Schwob, a35.1	333	104	8	221	0	0.93 [0.86, 0.97]	1.00 [0.98, 1.00]
Lindner, a53.1	179	30	11	137	1	0.73 [0.57, 0.86]	0.99 [0.96, 1.00]
Olearo, a54.1	184	41	43	100	0	0.49 [0.38, 0.60]	1.00 [0.96, 1.00]
Chaimao, a57.1	454	59	1	389	5	0.98 [0.91, 1.00]	0.99 [0.97, 1.00]
FINDdx, a64.1	400	94	12	287	7	0.89 [0.81, 0.94]	0.98 [0.95, 0.99]
Turcato, f09.1	3410	179	44	3157	30	0.80 [0.74, 0.85]	0.99 [0.99, 0.99]
Lindner, f15.1	146	34	6	105	1	0.85 [0.70, 0.94]	0.99 [0.95, 1.00]
Möckel, f19.1	271	67	22	182	0	0.75 [0.65, 0.84]	1.00 [0.98, 1.00]
Möckel, f19.2	202	18	7	176	1	0.72 [0.51, 0.88]	0.99 [0.97, 1.00]
Osterman, f20.2	642	165	91	377	9	0.64 [0.58, 0.70]	0.98 [0.96, 0.99]
Kannian, f26.1	37	15	12	10	0	0.56 [0.35, 0.74]	1.00 [0.69, 1.00]
Kohmer, f32.2	100	32	42	26	0	0.43 [0.32, 0.55]	1.00 [0.87, 1.00]
Baro, f33.3	286	44	57	178	7	0.44 [0.34, 0.54]	0.96 [0.92, 0.98]
Caruana, f34.1	532	47	67	417	1	0.41 [0.32, 0.51]	1.00 [0.99, 1.00]
Nikolai, f35.3	96	31	3	62	0	0.91 [0.76, 0.98]	1.00 [0.94, 1.00]
Pena, f36.1	842	51	22	766	3	0.70 [0.58, 0.80]	1.00 [0.99, 1.00]
Ristic, f44.1	120	25	18	77	0	0.58 [0.42, 0.73]	1.00 [0.95, 1.00]
Jääskeläinen, f50.2	198	128	30	40	0	0.81 [0.74, 0.87]	1.00 [0.91, 1.00]
Salvagno, f54.1	321	108	41	171	1	0.72 [0.65, 0.80]	0.99 [0.97, 1.00]
Schuit, f64.2	1596	83	49	1456	8	0.63 [0.54, 0.71]	1.00 [0.99, 1.00]
Kernéis, f69.1	1109	81	5	1013	10	0.94 [0.87, 0.98]	0.99 [0.98, 1.00]
Thell, f81.1	541	171	42	325	3	0.80 [0.74, 0.85]	0.99 [0.97, 1.00]
Homza, f87.4	139	26	16	96	1	0.62 [0.46, 0.76]	0.99 [0.94, 1.00]

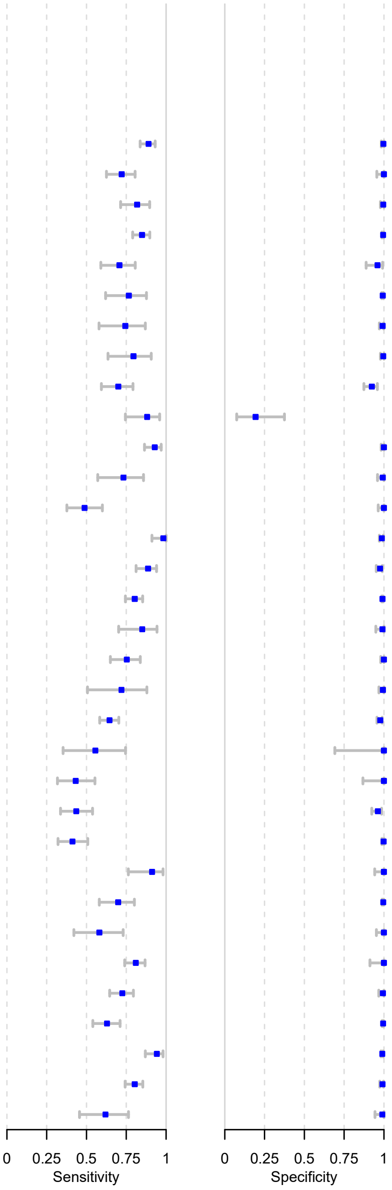

Standard F

Author, Study ID	Sample size	TP	FN	TN	FP	Sensitivity [95%CI]	Specificity [95%CI]
Drevinek, a10.2	591	139	84	366	2	0.62 [0.56, 0.69]	1.00 [0.98, 1.00]
Liotti, a22.1	359	49	55	251	4	0.47 [0.37, 0.57]	0.98 [0.96, 1.00]
Porte, a32.2	64	29	3	31	1	0.91 [0.75, 0.98]	0.97 [0.84, 1.00]
FINDdx, a63.1	453	93	27	326	7	0.78 [0.69, 0.85]	0.98 [0.96, 0.99]
Osterman, f20.1	549	115	74	352	8	0.61 [0.54, 0.68]	0.98 [0.96, 0.99]
FINDdx, a63.2	676	27	12	617	20	0.69 [0.52, 0.83]	0.97 [0.95, 0.98]

Coris

Author, Study ID	Sample size	TP	FN	TN	FP	Sensitivity [95%CI]	Specificity [95%CI]
Lambert−Niclot, a18.1	138	47	47	44	0	0.50 [0.40, 0.60]	1.00 [0.92, 1.00]
Scohy, a34.1	148	32	74	42	0	0.30 [0.22, 0.40]	1.00 [0.92, 1.00]
Veyrenche, a40.1	65	13	32	20	0	0.29 [0.16, 0.44]	1.00 [0.83, 1.00]
Mertens, a48.1	328	76	56	195	1	0.58 [0.49, 0.66]	1.00 [0.97, 1.00]
Ciotti, f24.1	50	12	27	11	0	0.31 [0.17, 0.48]	1.00 [0.72, 1.00]

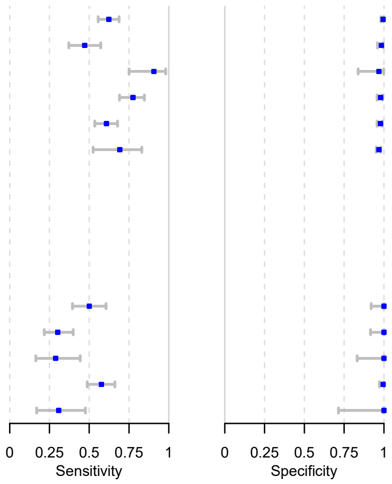

Standard Q nasal

Author, Study ID	Sample size	TP	FN	TN	FP	Sensitivity [95%CI]	Specificity [95%CI]
Lindner, f15.2	144	33	7	104	0	0.82 [0.67, 0.93]	1.00 [0.96, 1.00]
Nikolai, f35.1	132	31	5	96	0	0.86 [0.70, 0.95]	1.00 [0.96, 1.00]
Nikolai, f35.2	132	31	5	96	0	0.86 [0.70, 0.95]	1.00 [0.96, 1.00]
Nikolai, f35.4	96	31	3	61	1	0.91 [0.76, 0.98]	0.98 [0.91, 1.00]
Lindner, a53.2	179	33	8	136	2	0.80 [0.65, 0.91]	0.99 [0.95, 1.00]
Stohr, f45.2	1588	118	74	1392	4	0.62 [0.54, 0.68]	1.00 [0.99, 1.00]

Innova

Author, Study ID	Sample size	TP	FN	TN	FP	Sensitivity [95%CI]	Specificity [95%CI]
Houston, f25.1	728	242	38	426	22	0.86 [0.82, 0.90]	0.95 [0.93, 0.97]
Young, f56.1	786	133	81	572	0	0.62 [0.55, 0.69]	1.00 [0.99, 1.00]
Pickering, f73.1	200	89	11	99	1	0.89 [0.81, 0.94]	0.99 [0.95, 1.00]

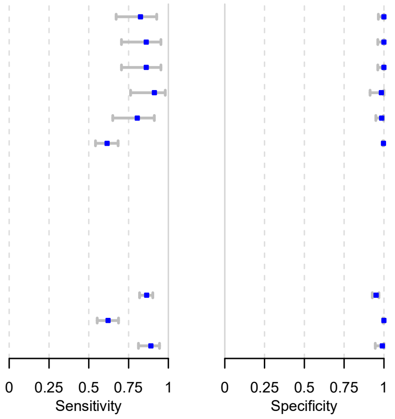

VIRO AAZ

Author, Study ID	Sample size	TP	FN	TN	FP	Sensitivity [95%CI]	Specificity [95%CI]
Schwob, a35.3	324	116	22	186	0	0.84 [0.77, 0.90]	1.00 [0.98, 1.00]

Savant

Author, Study ID	Sample size	TP	FN	TN	FP	Sensitivity [95%CI]	Specificity [95%CI]
Weitzel, a41.3	109	13	65	31	0	0.17 [0.09, 0.27]	1.00 [0.89, 1.00]

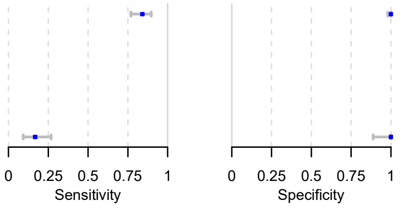

VIVADiag

Author, Study ID	Sample size	TP	FN	TN	FP	Sensitivity [95%CI]	Specificity [95%CI]
Homza, f87.5	268	38	53	170	7	0.42 [0.32, 0.53]	0.96 [0.92, 0.98]

ACCUCARE

Author, Study ID	Sample size	TP	FN	TN	FP	Sensitivity [95%CI]	Specificity [95%CI]
Thakur, f88.1	677	29	55	592	1	0.34 [0.24, 0.46]	1.00 [0.99, 1.00]

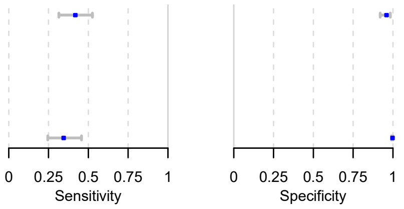

Supplement: S3 Fig — (PDF) [file pmed.1003735.s003.pdf]
