# Supplementary material for: Accuracy of novel antigen rapid diagnostics for SARS-CoV-2: A living systematic review and meta-analysis
Source: PLoS Med. 2021 Aug 12;18(8):e1003735. doi: 10.1371/journal.pmed.1003735 (PMC8389849; doi:10.1371/journal.pmed.1003735)

S4 Fig. Forest plots for subgroup analysis by CT-values.

Caption: CI = confidence interval

Fig A - Forest plot for CT values greater 20

Author, Study ID	Sample size	Sensitivity [95%CI]
Alemany, a02.ov	305	0.99 [0.98, 1.00]
Krüttgen, a16.1	12	1.00 [0.74, 1.00]
Takeda, a50.1	23	1.00 [0.85, 1.00]
Gupta, a13.1	28	0.86 [0.67, 0.96]
Bouassa, f67.1	52	0.94 [0.84, 0.99]
Sberna, f83.1	14	0.07 [0.00, 0.34]
Favresse, f31.1	22	0.86 [0.65, 0.97]
Favresse, f31.2	22	0.96 [0.77, 1.00]
Favresse, f31.3	22	0.96 [0.77, 1.00]
Favresse, f31.4	22	0.96 [0.77, 1.00]
Favresse, f31.5	22	1.00 [0.85, 1.00]
Young, f56.1	54	0.43 [0.29, 0.57]

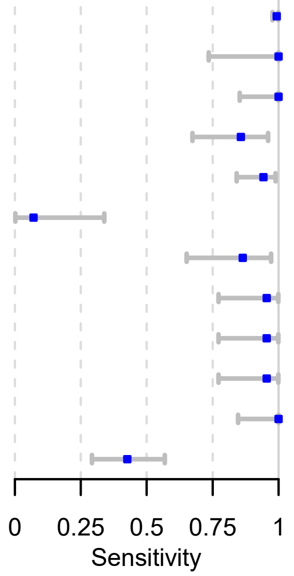

Fig B - Forest plot for CT values lower 20

Author, Study ID	Sample size	Sensitivity [95%CI]
Alemany, a02.ov	258	0.98 [0.96, 1.00]
Gupta, a13.1	5	0.80 [0.28, 1.00]
Krüttgen, a16.1	5	1.00 [0.48, 1.00]
Takeda, a50.1	9	1.00 [0.66, 1.00]
Gupta, a13.1	27	0.96 [0.81, 1.00]
Favresse, f31.1	2	1.00 [0.16, 1.00]
Favresse, f31.2	2	0.50 [0.01, 0.99]
Favresse, f31.3	2	1.00 [0.16, 1.00]
Favresse, f31.4	2	1.00 [0.16, 1.00]
Favresse, f31.5	2	1.00 [0.16, 1.00]
Pena, f36.1	31	0.97 [0.83, 1.00]
Young, f56.1	71	0.83 [0.72, 0.91]
Faíco−Filho, f63.1	13	1.00 [0.75, 1.00]
Bouassa, f67.1	25	1.00 [0.86, 1.00]
Thell, f81.1	51	1.00 [0.93, 1.00]
Sberna, f83.1	4	0.75 [0.19, 0.99]
Favresse, f31.1	34	0.97 [0.85, 1.00]
Favresse, f31.2	34	0.94 [0.80, 0.99]
Favresse, f31.3	34	0.97 [0.85, 1.00]
Favresse, f31.4	34	0.97 [0.85, 1.00]
Favresse, f31.5	34	1.00 [0.90, 1.00]
Young, f56.1	62	0.76 [0.63, 0.86]

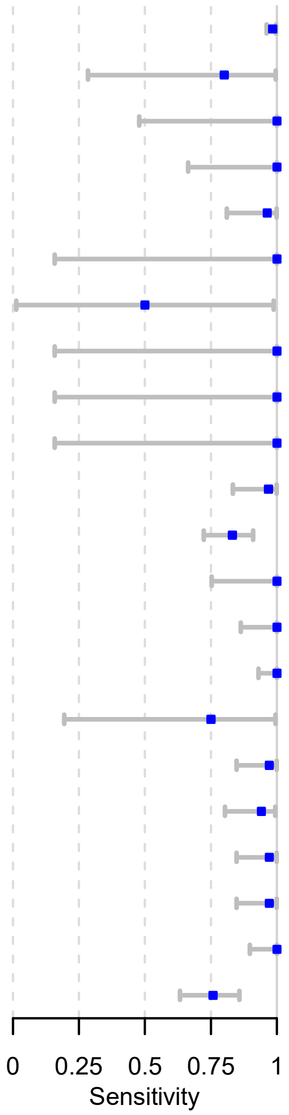

Fig C - Forest plot for CT values greater 25

Author, Study ID	Sample size	Sensitivity [95%CI]
Bulilete, a07.1	25	0.40 [0.21, 0.61]
Krüger, a17.1	29	0.62 [0.42, 0.79]
Krüger, a17.2	8	0.25 [0.03, 0.65]
Krüger, a17.3	5	0.40 [0.05, 0.85]
Weitzel, a41.1	26	0.15 [0.04, 0.35]
Weitzel, a41.3	26	0.08 [0.01, 0.25]
Weitzel, a41.4	26	0.54 [0.33, 0.73]
Porte, a31.1	18	0.72 [0.47, 0.90]
Krüger, a52.ov	33	0.67 [0.48, 0.82]
Alemany, a02.ov	285	0.90 [0.86, 0.93]
Takeda, a50.1	18	0.89 [0.65, 0.99]
Gupta, a13.1	13	0.62 [0.32, 0.86]
Jääskeläinen, f50.1	34	0.82 [0.66, 0.93]
Jääskeläinen, f50.2	35	0.69 [0.51, 0.83]
Jääskeläinen, f50.3	34	0.76 [0.59, 0.89]
Pérez−García, f52.1	86	0.14 [0.07, 0.23]
Pérez−García, f52.2	86	0.24 [0.16, 0.35]
Krüger, f58.1	51	0.63 [0.48, 0.76]
Sberna, f83.1	23	0.04 [0.00, 0.22]
Favresse, f31.1	19	0.32 [0.13, 0.57]
Favresse, f31.2	19	0.53 [0.29, 0.76]
Favresse, f31.3	19	0.68 [0.43, 0.87]
Favresse, f31.4	19	0.53 [0.29, 0.76]
Favresse, f31.5	19	1.00 [0.82, 1.00]
Young, f56.1	23	0.17 [0.05, 0.39]
Thakur, f88.1	21	0.10 [0.01, 0.30]

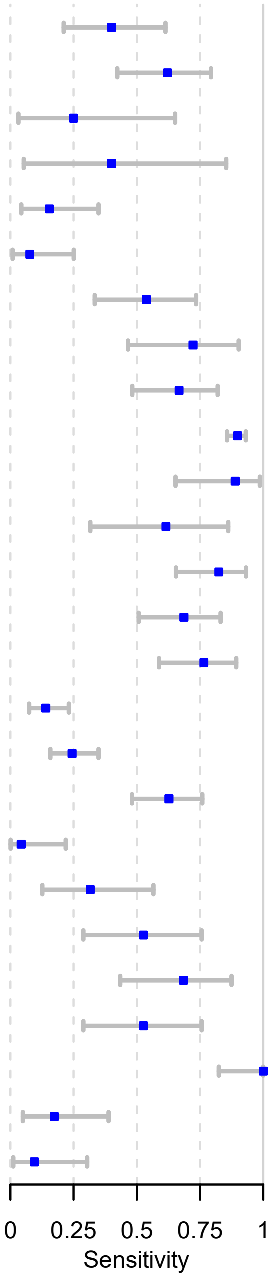

Fig D - Forest plot for CT values lower 25

Author, Study ID	Sample size	Sensitivity [95%CI]
Bulilete, a07.1	95	0.90 [0.81, 0.95]
Krüger, a17.1	18	1.00 [0.81, 1.00]
Weitzel, a41.1	53	0.85 [0.72, 0.93]
Weitzel, a41.3	52	0.21 [0.11, 0.35]
Weitzel, a41.4	54	1.00 [0.93, 1.00]
Porte, a31.1	52	1.00 [0.93, 1.00]
Porte, a32.1	27	1.00 [0.87, 1.00]
Porte, a32.2	27	1.00 [0.87, 1.00]
Scohy, a34.1	10	1.00 [0.69, 1.00]
Krüger, a52.ov	72	0.96 [0.88, 0.99]
Agullo, a56.2	68	0.75 [0.63, 0.85]
Agullo, a56.3	63	0.38 [0.26, 0.51]
Alemany, a02.ov	305	0.99 [0.98, 1.00]
Krüttgen, a16.1	12	1.00 [0.74, 1.00]
Takeda, a50.1	23	1.00 [0.85, 1.00]
Gupta, a13.1	28	0.86 [0.67, 0.96]
Strömer, f11.1	32	1.00 [0.89, 1.00]
Pollock, f17.1	150	0.99 [0.96, 1.00]
Jääskeläinen, f50.1	89	0.99 [0.94, 1.00]
Jääskeläinen, f50.2	97	0.99 [0.94, 1.00]
Jääskeläinen, f50.3	92	0.98 [0.92, 1.00]
Pérez−García, f52.1	84	0.94 [0.87, 0.98]
Pérez−García, f52.2	84	0.96 [0.90, 0.99]
Krüger, f58.1	95	0.93 [0.85, 0.97]
Pollock, f59.1	95	0.96 [0.90, 0.99]
Pollock, f59.1	12	1.00 [0.74, 1.00]

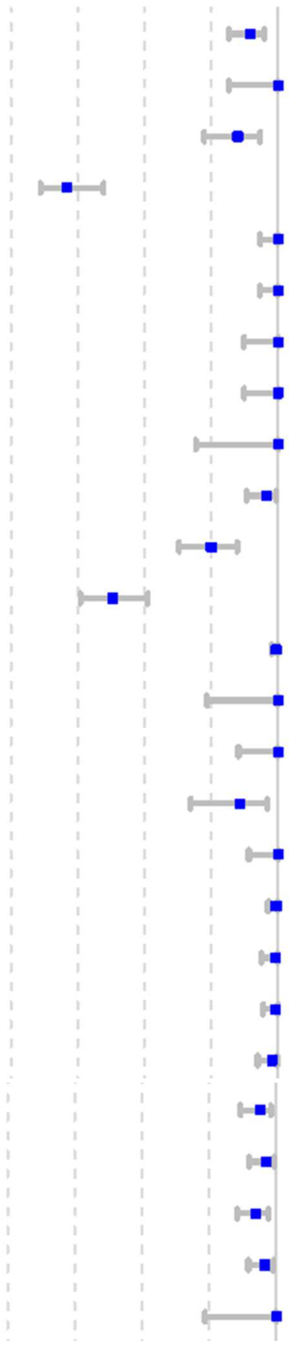

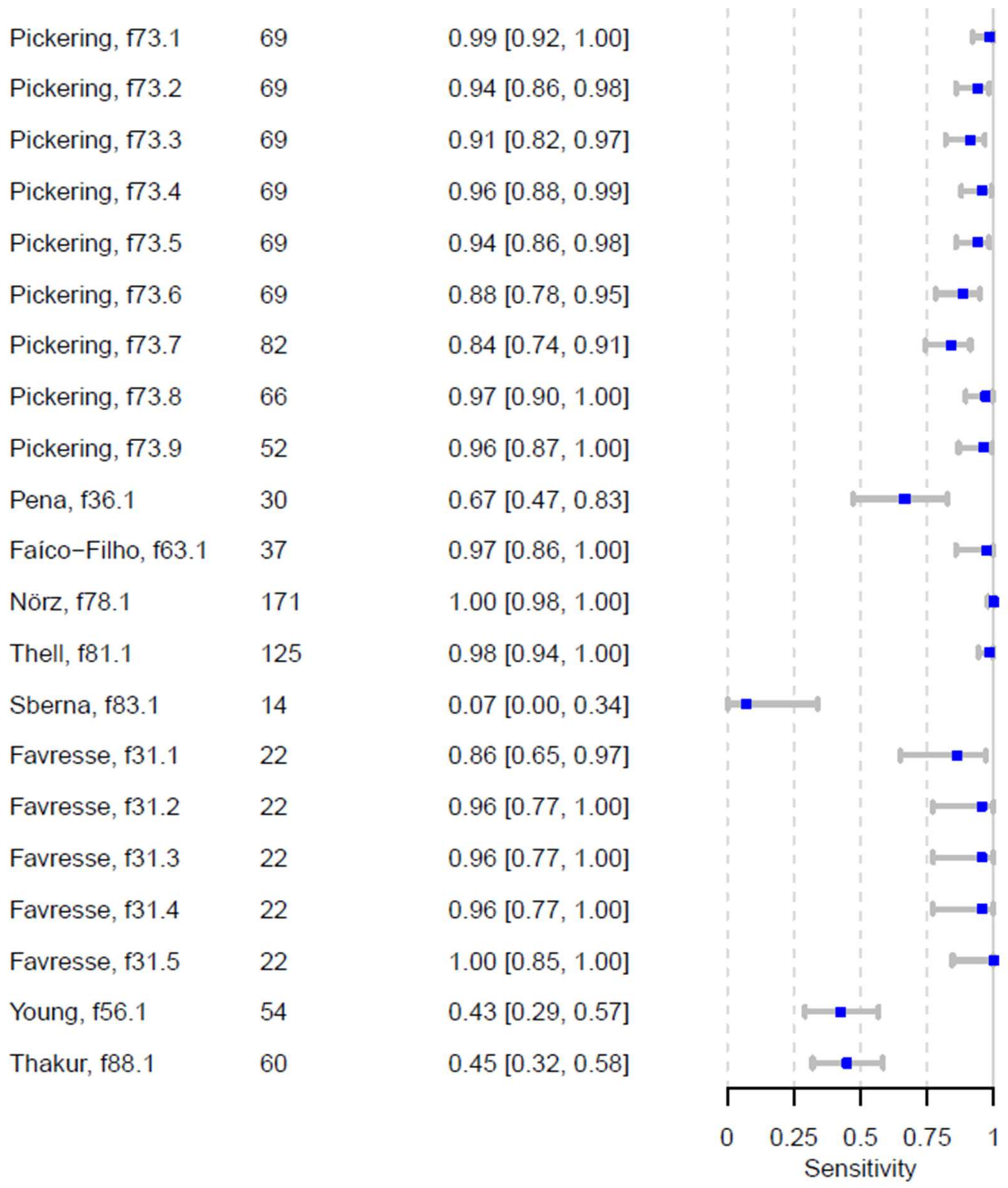
Pickering, f73.1 69 0.99 [0.92, 1.00]
Pickering, f73.2 69 0.94 [0.86, 0.98]
Pickering, f73.3 69 0.91 [0.82, 0.97]
Pickering, f73.4 69 0.96 [0.88, 0.99]
Pickering, f73.5 69 0.94 [0.86, 0.98]
Pickering, f73.6 69 0.88 [0.78, 0.95]
Pickering, f73.7 82 0.84 [0.74, 0.91]
Pickering, f73.8 66 0.97 [0.90, 1.00]
Pickering, f73.9 52 0.96 [0.87, 1.00]
Pena, f36.1 30 0.67 [0.47, 0.83]
Faíco−Filho, f63.1 37 0.97 [0.86, 1.00]
Nörz, f78.1 171 1.00 [0.98, 1.00]
Thell, f81.1 125 0.98 [0.94, 1.00]
Sberna, f83.1 14 0.07 [0.00, 0.34]
Favresse, f31.1 22 0.86 [0.65, 0.97]
Favresse, f31.2 22 0.96 [0.77, 1.00]
Favresse, f31.3 22 0.96 [0.77, 1.00]
Favresse, f31.4 22 0.96 [0.77, 1.00]
Favresse, f31.5 22 1.00 [0.85, 1.00]
Young, f56.1 54 0.43 [0.29, 0.57]
Thakur, f88.1 60 0.45 [0.32, 0.58]
0 0.25 0.5 0.75 1
Sensitivity

Fig E - forest plot for CT values greater 30

Author, Study ID	Sample size	Sensitivity [95%CI]
Merino−Amador, a25.1	41	0.51 [0.35, 0.67]
Nalumansi, a27.1	44	0.54 [0.39, 0.70]
Bulilete, a07.1	14	0.14 [0.02, 0.43]
Nalumansi, a27.1	9	0.56 [0.21, 0.86]
Alemany, a02.ov	103	0.57 [0.47, 0.67]
Takeda, a50.1	12	0.17 [0.02, 0.48]
Krüger, a52.ov	12	0.33 [0.10, 0.65]
Gupta, a13.1	4	0.25 [0.01, 0.81]
Pena, f36.1	12	0.08 [0.00, 0.38]
Bouassa, f67.1	23	0.70 [0.47, 0.87]
Jääskeläinen, f50.1	25	0.12 [0.03, 0.31]
Jääskeläinen, f50.2	26	0.31 [0.14, 0.52]
Jääskeläinen, f50.3	26	0.38 [0.20, 0.59]
Okoye, f51.1	9	0.00 [0.00, 0.34]
Krüger, f58.1	24	0.42 [0.22, 0.63]
Sberna, f83.1	21	0.00 [0.00, 0.16]
Favresse, f31.1	3	0.33 [0.01, 0.91]
Favresse, f31.2	3	0.00 [0.00, 0.71]
Favresse, f31.3	3	0.67 [0.09, 0.99]
Favresse, f31.4	3	0.00 [0.00, 0.71]
Favresse, f31.5	3	1.00 [0.29, 1.00]
Young, f56.1	3	0.00 [0.00, 0.71]
Favresse, f31.1	16	0.19 [0.04, 0.46]
Favresse, f31.2	16	0.06 [0.00, 0.30]
Favresse, f31.3	16	0.19 [0.04, 0.46]
Favresse, f31.4	16	0.06 [0.00, 0.30]
Favresse, f31.5	16	0.00 [0.00, 0.21]
Okoye, f51.1	3	0.00 [0.00, 0.71]
Thakur, f88.1	3	0.00 [0.00, 0.71]

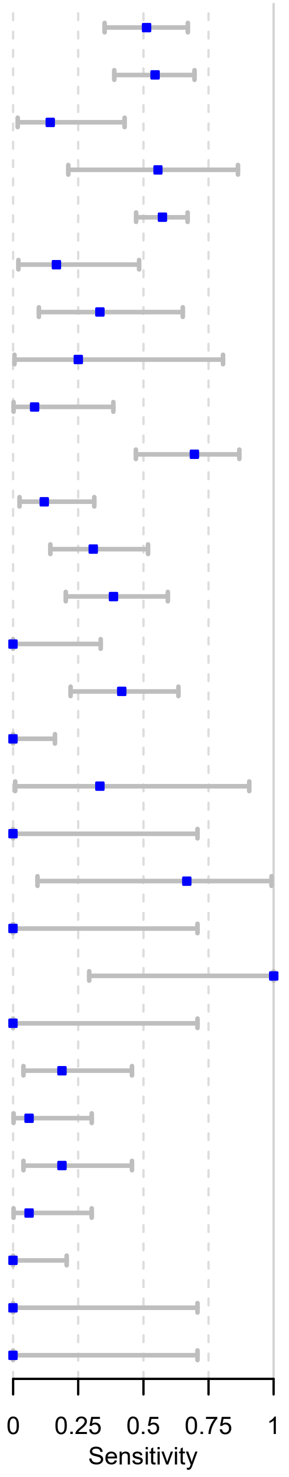

Fig F - forest plot for CT value lower 30

Author, Study ID	Sample size	Sensitivity [95%CI]
Merino−Amador, a25.1	41	0.51 [0.35, 0.67]
Nalumansi, a27.1	44	0.54 [0.39, 0.70]
Bulilete, a07.1	14	0.14 [0.02, 0.43]
Nalumansi, a27.1	9	0.56 [0.21, 0.86]
Alemany, a02.ov	103	0.57 [0.47, 0.67]
Takeda, a50.1	12	0.17 [0.02, 0.48]
Krüger, a52.ov	12	0.33 [0.10, 0.65]
Gupta, a13.1	4	0.25 [0.01, 0.81]
Pena, f36.1	12	0.08 [0.00, 0.38]
Bouassa, f67.1	23	0.70 [0.47, 0.87]
Jääskeläinen, f50.1	25	0.12 [0.03, 0.31]
Jääskeläinen, f50.2	26	0.31 [0.14, 0.52]
Jääskeläinen, f50.3	26	0.38 [0.20, 0.59]
Okoye, f51.1	9	0.00 [0.00, 0.34]
Krüger, f58.1	24	0.42 [0.22, 0.63]
Sberna, f83.1	21	0.00 [0.00, 0.16]
Favresse, f31.1	3	0.33 [0.01, 0.91]
Favresse, f31.2	3	0.00 [0.00, 0.71]
Favresse, f31.3	3	0.67 [0.09, 0.99]
Favresse, f31.4	3	0.00 [0.00, 0.71]
Favresse, f31.5	3	1.00 [0.29, 1.00]
Young, f56.1	3	0.00 [0.00, 0.71]
Favresse, f31.1	16	0.19 [0.04, 0.46]
Favresse, f31.2	16	0.06 [0.00, 0.30]
Favresse, f31.3	16	0.19 [0.04, 0.46]
Favresse, f31.4	16	0.06 [0.00, 0.30]
Favresse, f31.5	16	0.00 [0.00, 0.21]
Okoye, f51.1	3	0.00 [0.00, 0.71]
Thakur, f88.1	3	0.00 [0.00, 0.71]

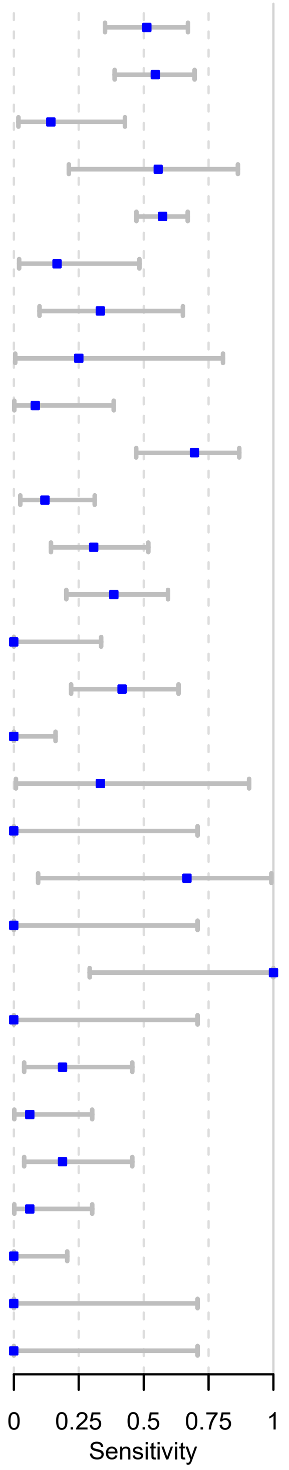

Supplement: S4 Fig — (PDF) [file pmed.1003735.s004.pdf]
