# Supplementary material for: Accuracy of novel antigen rapid diagnostics for SARS-CoV-2: A living systematic review and meta-analysis
Source: PLoS Med. 2021 Aug 12;18(8):e1003735. doi: 10.1371/journal.pmed.1003735 (PMC8389849; doi:10.1371/journal.pmed.1003735)

S5 Fig. Forest plots for subgroup analysis by CT-values per test.

Caption: CI = confidence interval

Fig A - Forest plot for CT values lower 20

Test assessed	N datasets	Total Sample Size	Pooled Sensitivity (95% CI)
Panbio	4	307	97.7% (95.3−98.9)
Standard Q	5	119	97.5% (92.5−99.2)

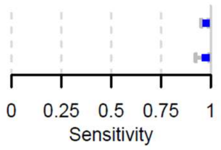

Fig B - Forest plot for CT values greater 25

Test assessed	N datasets	Total Sample Size	Pooled Sensitivity (95% CI)
Panbio	6	482	61.2% (38.8−79.7)

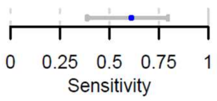

Fig C - Forest plot for CT values lower 25

Test assessed	N datasets	Total Sample Size	Pooled Sensitivity (95% CI)
Panbio	9	838	94.1% (83.8−98)
Standard Q	6	310	96.7% (84.4−99.4)

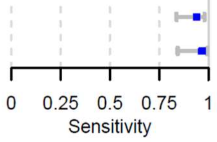

Fig D - forest plot for CT values greater 30

Test assessed	N datasets	Total Sample Size	Pooled Sensitivity (95% CI)
Panbio	7	215	30.5% (16−50.4)
Standard Q	5	95	35.8% (20.1−55.2)

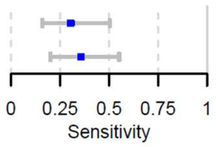

Fig E - forest plot for CT value lower 30

Test assessed	N datasets	Total Sample Size	Pooled Sensitivity (95% CI)
BinaxNOW	5	323	86.3% (66.5−95.3)
Panbio	12	1233	83.4% (69.1−91.9)
Standard Q	5	390	85.7% (73.8−92.7)

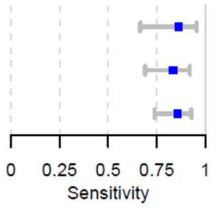

Supplement: S5 Fig — (PDF) [file pmed.1003735.s005.pdf]
