# Supplementary material for: Accuracy of novel antigen rapid diagnostics for SARS-CoV-2: A living systematic review and meta-analysis
Source: PLoS Med. 2021 Aug 12;18(8):e1003735. doi: 10.1371/journal.pmed.1003735 (PMC8389849; doi:10.1371/journal.pmed.1003735)

S8 Fig. Forest plots for subgroup analysis by symptomatic vs. asymptomatic.

Caption: CI = confidence interval

Figure A - Forest plot of asymptomatic patients

**Author, Study ID**	**Sample size**	**Sensitivity [95%CI]**
Alemany, a02.ov	117	0.80 [0.71, 0.86]
Fenollar. F., a11.1	22	0.46 [0.24, 0.68]
Gupta, a13.1	13	0.69 [0.39, 0.91]
Merino−Amador, a25.1	31	1.00 [0.89, 1.00]
Scohy, a34.1	14	0.29 [0.08, 0.58]
Shrestha, a36.1	47	0.85 [0.72, 0.94]
Torres, a37.1	79	0.48 [0.37, 0.60]
Krüger, a52.ov	14	0.86 [0.57, 0.98]
Agullo, a56.2	35	0.23 [0.10, 0.40]
Agullo, a56.3	32	0.09 [0.02, 0.25]
Parada−Ricart, a58.1	1	0.00 [0.00, 0.98]
Turcato, f09.1	54	0.50 [0.36, 0.64]
Pollock, f17.1	107	0.65 [0.56, 0.74]
Pollock, f17.1	57	0.70 [0.57, 0.82]
James, f23.1	128	0.52 [0.43, 0.60]
Baro, f33.1	101	0.39 [0.29, 0.49]
Baro, f33.2	101	0.52 [0.41, 0.62]
Baro, f33.3	101	0.44 [0.34, 0.54]
Baro, f33.4	101	0.46 [0.36, 0.56]
Baro, f33.5	101	0.29 [0.20, 0.39]
Pena, f36.1	73	0.70 [0.58, 0.80]
Okoye, f51.1	45	0.53 [0.38, 0.68]
Krüger, f58.1	9	0.78 [0.40, 0.97]
L...Huillier, f72.1	30	0.43 [0.26, 0.63]
Nörz, f78.1	79	0.28 [0.18, 0.39]
Thakur, f88.1	84	0.34 [0.24, 0.46]

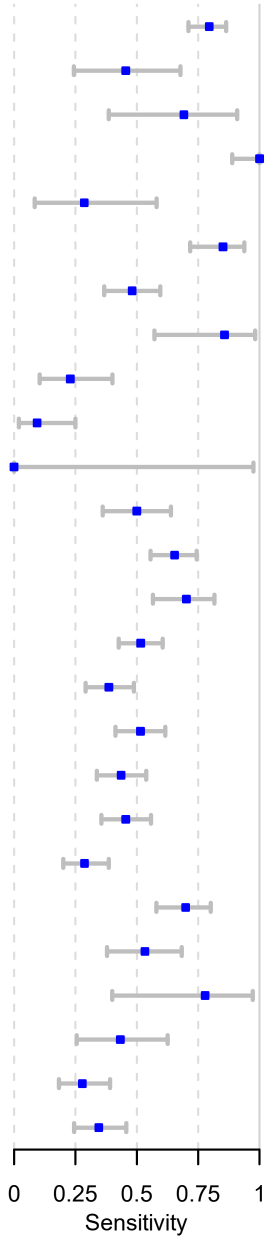

Figure B - Forest plot of symptomatic patients

Author, Study ID	Sample size	Sensitivity [95%CI]
Abdulrahman, a01.1	733	0.82 [0.79, 0.85]
Alemany, a02.ov	419	0.93 [0.90, 0.95]
Albert, a03.1	54	0.80 [0.66, 0.89]
Beck, a04.1	61	0.77 [0.64, 0.87]
Cerutti, a08.1	104	0.72 [0.62, 0.80]
Fenollar. F., a11.1	182	0.79 [0.72, 0.85]
Gupta, a13.1	64	0.84 [0.73, 0.92]
Merino−Amador, a25.1	328	0.90 [0.86, 0.93]
Pekosz, a28.1	28	0.96 [0.82, 1.00]
Porte, a31.1	82	0.94 [0.86, 0.98]
Scohy, a34.1	77	0.32 [0.22, 0.44]
Schwob, a35.1	112	0.93 [0.86, 0.97]
Schwob, a35.2	122	0.86 [0.79, 0.92]
Schwob, a35.3	138	0.84 [0.77, 0.90]
Weitzel, a41.1	79	0.62 [0.50, 0.73]
Weitzel, a41.2	9	0.00 [0.00, 0.34]
Weitzel, a41.3	78	0.17 [0.09, 0.27]
Weitzel, a41.4	80	0.85 [0.75, 0.92]
Young, a43.1	38	0.76 [0.60, 0.89]
Herrera, a46.1	459	0.77 [0.72, 0.80]
Krüger, a52.ov	91	0.87 [0.78, 0.93]
Lindner, a53.1	41	0.73 [0.57, 0.86]
Agullo, a56.2	97	0.53 [0.42, 0.63]
Agullo, a56.3	89	0.28 [0.19, 0.39]
Parada−Ricart, a58.1	25	0.76 [0.55, 0.91]
Turcato, f09.1	169	0.90 [0.84, 0.94]
Basso, f10.1	87	0.82 [0.72, 0.89]

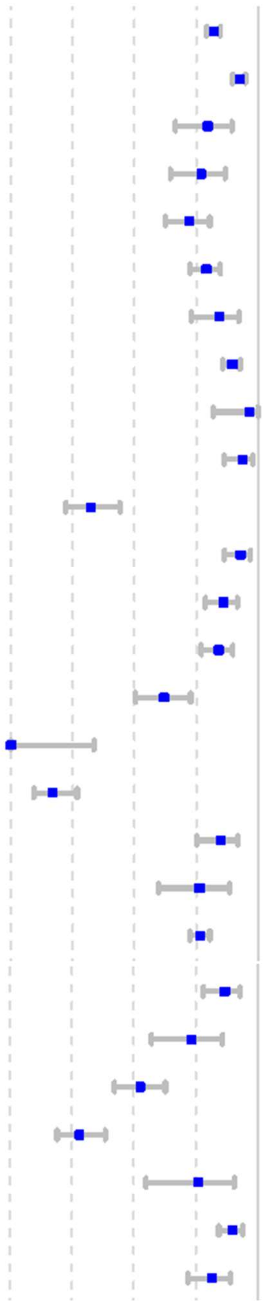

Takeuchi, f12.1	72	0.92 [0.83, 0.97]
Lindner, f15.1	40	0.85 [0.70, 0.94]
Lindner, f15.2	40	0.82 [0.67, 0.93]
Pollock, f17.1	128	0.91 [0.84, 0.95]
Möckel, f19.1	89	0.75 [0.65, 0.84]
Möckel, f19.2	25	0.72 [0.51, 0.88]
James, f23.1	24	0.83 [0.63, 0.95]
Nikolai, f35.1	36	0.86 [0.70, 0.95]
Nikolai, f35.2	36	0.86 [0.70, 0.95]
Nikolai, f35.3	34	0.91 [0.76, 0.98]
Nikolai, f35.4	34	0.91 [0.76, 0.98]
Lindner, a53.2	41	0.80 [0.65, 0.91]
Ristic, f44.1	43	0.58 [0.42, 0.73]
Villaverde, f55.1	77	0.46 [0.34, 0.57]
Krüger, f58.1	53	0.85 [0.72, 0.93]
Shidlovskaya, f61.1	78	0.56 [0.45, 0.68]
Shidlovskaya, f61.2	78	0.53 [0.41, 0.64]
Bouassa, f67.1	100	0.90 [0.82, 0.95]
L...Huillier, f72.1	89	0.73 [0.63, 0.82]
Nörz, f78.1	313	0.68 [0.63, 0.74]
Osmanodja, f79.1	70	0.89 [0.79, 0.95]
Thell, f81.1	213	0.80 [0.74, 0.85]

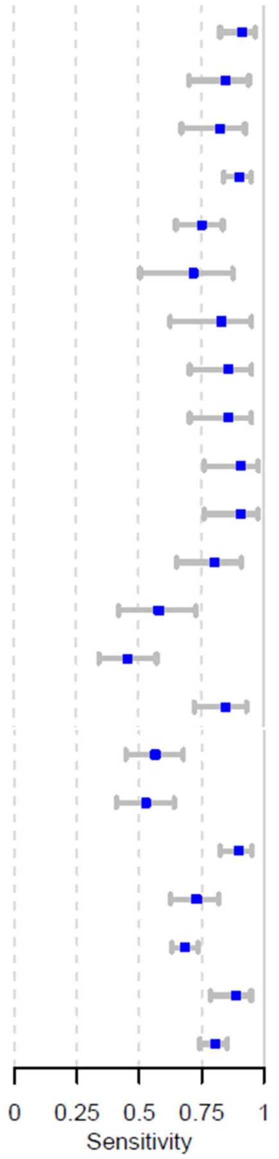

Supplement: S8 Fig — (PDF) [file pmed.1003735.s008.pdf]
