# Supplementary material for: Accuracy of novel antigen rapid diagnostics for SARS-CoV-2: A living systematic review and meta-analysis
Source: PLoS Med. 2021 Aug 12;18(8):e1003735. doi: 10.1371/journal.pmed.1003735 (PMC8389849; doi:10.1371/journal.pmed.1003735)

S9 Fig. Forest plots for subgroup analysis by symptom duration.

Caption: CI = confidence interval

Figure A - Forest plot of patients with symptom onset greater than seven days

Author, Study ID	Sample size	Sensitivity [95%CI]
Beck, a04.1	11	0.54 [0.23, 0.83]
Gupta, a13.1	7	0.71 [0.29, 0.96]
Krüger, a17.1	4	1.00 [0.40, 1.00]
Linares, a20.1	13	0.54 [0.25, 0.81]
Porte, a31.1	5	0.80 [0.28, 1.00]
Krüger, a52.ov	13	0.62 [0.32, 0.86]
Pollock, f17.1	2	1.00 [0.16, 1.00]
Pollock, f17.1	15	0.67 [0.38, 0.88]
Drain, f43.1	10	0.90 [0.56, 1.00]
Drain, f43.2	6	0.83 [0.36, 1.00]
Pérez−García, f52.1	55	0.49 [0.35, 0.63]
Pérez−García, f52.2	55	0.58 [0.44, 0.71]
Krüger, f58.1	13	0.54 [0.25, 0.81]
Shidlovskaya, f61.1	45	0.36 [0.22, 0.51]
Shidlovskaya, f61.2	45	0.31 [0.18, 0.47]
Nörz, f78.1	109	0.46 [0.36, 0.56]
Thell, f81.1	24	0.62 [0.41, 0.81]

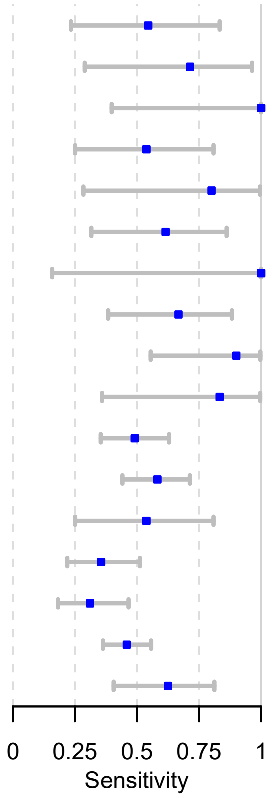

Figure B - Forest plot of patients with symptom onset less than seven days

Author, Study ID	Sample size	Sensitivity [95%CI]
Beck, a04.1	50	0.82 [0.69, 0.91]
Gupta, a13.1	57	0.86 [0.74, 0.94]
Krüger, a17.1	35	0.80 [0.63, 0.92]
Linares, a20.1	37	0.86 [0.71, 0.96]
Porte, a31.1	76	0.95 [0.87, 0.98]
Young, a43.1	38	0.76 [0.60, 0.89]
Krüger, a52.ov	76	0.91 [0.82, 0.96]
Pollock, f17.1	26	0.85 [0.65, 0.96]
Pollock, f17.1	85	0.96 [0.90, 0.99]
Drain, f43.1	73	0.99 [0.93, 1.00]
Drain, f43.2	34	1.00 [0.90, 1.00]
Pérez−García, f52.1	46	0.85 [0.71, 0.94]
Pérez−García, f52.2	46	0.91 [0.79, 0.98]
Villaverde, f55.1	77	0.46 [0.34, 0.57]
Krüger, f58.1	118	0.86 [0.79, 0.92]
Shidlovskaya, f61.1	43	0.65 [0.49, 0.79]
Shidlovskaya, f61.2	43	0.63 [0.47, 0.77]
Nörz, f78.1	149	0.83 [0.76, 0.89]
Thell, f81.1	189	0.82 [0.76, 0.88]

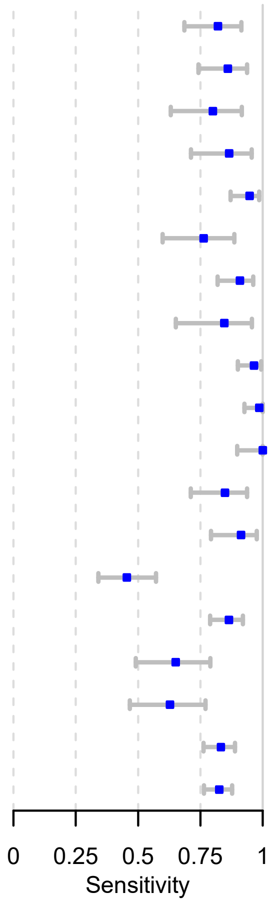

Supplement: S9 Fig — (PDF) [file pmed.1003735.s009.pdf]
