# Supplementary material for: Accuracy of novel antigen rapid diagnostics for SARS-CoV-2: A living systematic review and meta-analysis
Source: PLoS Med. 2021 Aug 12;18(8):e1003735. doi: 10.1371/journal.pmed.1003735 (PMC8389849; doi:10.1371/journal.pmed.1003735)
Supplement: Attachment — Submitted filename: Rebuttal Letter AgRDT SR_MA_v5.docx [file pmed.1003735.s018.docx]

Rebuttal Letter

**The accuracy of novel antigen rapid diagnostics for SARS-CoV-2: a living systematic review and meta-analysis.**

Editors comment:

| Please update your search to the end of March 2021, say. | We were able to update the analysis until the end of April, 2021 as we had already performed the extraction as part of our living systematic review. |
| --- | --- |
| Please remove the "Summary" on the title page (this could be repurposed in the author summary, below). | We have removed this section and incorporated it into the author summary (line 66 to 96). |
| Please combine the "Methods" and "Findings" subsections of your abstract. | Both subsections are now combined (line 39 to 60). |
| Please add a new final sentence to the combined subsection, which should begin "Study limitations include ..." or similar and should quote 2-3 of the study's main limitations. | We have added the two major limitations of our study at the end of the “Methods and Findings” subsection (line 59/60). |
| At line 61, please adapt the style of the "Conclusions" subsection of your abstract as follows: "In this study, we found that ... detected most cases ...", or similar. | We have rephrased the conclusion statement accordingly (line 61). |
| After the abstract, we will need to ask you to add a new and accessible "Author Summary" section in non-identical prose. You may find it helpful to consult one or two recent research papers in PLOS Medicine to get a sense of the preferred style. | We have added an “author summary” on the page following the abstract (line 66 to 96). |
| Noting the "living" element quoted in your title, we would suggest moving the references to the website, currently at line 105 and in the Methods, to a single mention in the Discussion section. Here we suggest noting how frequently the data will be updated. | We followed your suggestion and moved the refences to the website to the discussion (line 599 to 604). We did leave one reference each in the author summary (line 78) and introduction (line 131/132). We have also mentioned that we will update the website once a week and plan to update the MA on a monthly basis (line 599 to 604). |
| Please also explain to the editors how you plan to proceed with any future peer-reviewed analyses of these data. | We have not fully decided on this aspect yet and would be very grateful for your input. Would it be of interest to PLOS Med to publish an update (in a short format) later this year? We could also then include meta-regression and potentially additional sub-analyses by self- versus professional sampling as well as PCR versus viral culture as a comparison. |
| Throughout the text, please adapt the style of reference call-outs as follows: "...[12,13]." (noting the absence of spaces within the square brackets). | We have adapted the style of references accordingly. |
| Noting references 17 and 31, for example, please ensure that all citations have full access details. | We have checked all sources again to ensure they provide sufficient information (line 627 to line 1599). |
| Noting the preprints cited, can DOI numbers, for example, be added so that these can be accessed easily? | We have added DOI numbers to all sources (line 627 to line 1599). |
| Thank you for including the PRISMA checklist. May we suggest using PRISMA 2020 (<https://doi.org/10.1371/journal.pmed.1003583>) in future? | Thank you for referring to the updated PRISMA checklist. We have adopted this version for the paper. |
| Please break the checklist out into a separate attachment, labelled "S1_PRISMA_Checklist" or similar and referred to as such in the main text. | We have submitted two different supplements: one labelled “S1_PRISMA_Checklist”, including only the checklist and one labelled “Supplements” including all other supplements (S2 – S15). The “S1_PRISMA_Checklist” is also referred to accordingly in the methods (line 135/136) |
| In the checklist, please refer to individual items by section (e.g., "Methods") and paragraph number, not by line or page numbers as these generally change in the event if publication. | We have adjusted the PRISMA checklist accordingly. |

Reviewer #1

| Estimated Authors,  I've read with great interest your paper on the accuracy of novel antigen rapid diagnostics for SARS-CoV-2.  The paper is well written, and addresses both the potential pros and the actuans cons of these new instruments.  Materials are clear, tables and images included in the main text are appropriate.  Discussion is consistent with the results and analyzes available evidence.  I've no further recommendations and suggest the eventual acceptance of this paper. | Thank you very much for this comment.  We really appreciate the thoughtful review. |
| --- | --- |

Reviewer #2, Alex McConnachie, Statistical Review

Brummer et al report a systematic review and meta-analysis of the diagnostic accuracy of rapid antigen tests for SARS-CoV-2. This review considers the use of statistics in the paper.

| Overall, this is very impressive. It reads quite well, and seems to cover most of the elements that are required. My comments are relatively minor. | We really appreciate your comment and are thankful for the review. |
| --- | --- |
| Line 384 makes a statement that the confidence intervals for the pooled sensitivity in symptomatic and asymptomatic patients are overlapping. I do not like this - overlapping Cis does not necessarily mean that there is no evidence of a difference between the two groups. It would be better to report a p-value, or a confidence interval for the difference in sensitivity. The same principle applies to other subgroup analyses - when looking at subgroups, I want to know whether there is a difference in the quantity being estimated (e.g. sensitivity) between the subgroups. | Thank you for pointing out this aspect. However, based on recent guidance (Rothmann Eur J Epidemiology 2016, Amrheim Commentary Nature 2019), we would like to avoid using p-values. In addition, due to the large amount of new data included in the revised version, the confidence intervals have narrowed making them more easily interpretable. |
| The subheading on line 408 mentions viral load, though the subgroup analysis by viral load is reported in the previous section. | We have adjusted the paragraph accordingly (line 501 to line 551). |
| Lines 423-426 mention publication bias, but this is very brief. P-values are given, but no interpretation. In what way are the results biased? Can the pooled estimates be corrected for this bias in some way? How does this affect the overall results? | We added an interpretation of the p-values in the results section (line 517 to line 522). |
| Finally, the forest plots do not include pooled estimates, or measures of heterogeneity. Would these be worth showing here? | Thank you for this suggestion! We have considered this carefully, but based on the Cochrane guidance for the reporting of diagnostic test accuracy (Macaskill P, Gatsonis C, Deeks JJ, Harbord RM, Takwoingi Y. Chapter  10: Analysing and Presenting Results. In: Deeks JJ, Bossuyt PM, Gatsonis C (editors), Cochrane  Handbook for Systematic Reviews of Diagnostic Test Accuracy Version 1.0. The Cochrane  Collaboration, 2010. Available from: <http://srdta.cochrane.org/>.), we would prefer not to present measures for heterogeneity. |

Reviewer #3

Review of the paper entitled

The accuracy of novel antigen rapid diagnostics for SARS-CoV-2: a living systematic review and meta-analysis.

General comment:

| The topic of this article is important as there is a great need to follow the improvement in the quality of lateral-flow rapid antigen tests for COVID-19, as it has been done for malaria rapid tests by FIND and WHO and other infectious diseases rapid tests by WHO (HIV, HBV..). Having a living review is very innovative and welcome.  For such an entreprise, there is a need for a well defined reference method that allows to comparing one brand or test version to another. This method can be based either on samples of patients with a pre-defined distribution (or at least a pre-defined threshold) of viral loads measured by quantitative PCR or on laboratory prepared samples containing defined concentration of antigen. No international standard for the validation of COVID RDT is unfortunately available yet. Therefore, the present review has to target studies that include patients whose viral load distribution is very variable from one study to the other, which limits strongly the comparability between studies and the interpretation of pooled results. Stratifying results by viral load thresholds, as authors have done, is therefore key and the main results should be these stratified results (using also a threshold of <20 would have been ideal) rather than the overall result. | Thank you very much for your detailed review and comments to our paper. We fully agree that the results by Ct-value should be presented more prominently and described below how we have integrated your comments. However, given that the number of studies that report on Ct-values in a comparable way are limited, we decided to leave the overall estimate as the main outcome. |
| --- | --- |

Specific comments:

**Abstract:**

| In "Results", in addition to the pooled sensitivity for Ct-values <30 (significant viral loads), the pooled sensitivity for Ct<25 (moderate and high viral loads) should be added, as it is an important result as mentioned above under general Comment, mentioned by the way by the authors in "Methods". If available, it would be good to also have the result for Ct<20 (high viral loads). | We have aligned the abstract in that we report sensitivity for <20 and <25, as well as for ≥25 and ≥30 (line 52/53). |
| --- | --- |
| In "Results", in order to show the performance of RDTs in usual testing conditions, e.g. using the good quality brands (SD Biosensor/Roche or Abbott), on the best sample type (naso-pharyngeal), during the first week of symptom (vast majority of patients seen in testing centers), it would be good to add the pooled sensitivity found in the corresponding relevant studies, if feasible. | Thank you for this suggestion. We agree with the importance of this analysis. Unfortunately, there is a limited amount of data that can be analysed combining the subgroup ‘duration of symptoms’ for those studies presenting data ‘IFU-conforming’. Therefore, we cannot present pooled estimates for IFU-conforming testing in the first week after symptom onset. We hope to be able to do this in the next iteration of the meta-analysis and will then present it on the website. |
| In "Conclusion", replace the word "screening" by "diagnosis" as you speak here about symptomatic patients. Also RDT do detect more than "most cases with high viral load". Indeed, they detect most cases with any viral load and the vast majority of patients with significant viral load (a viral load equivalent to a CT of 29 is not a high viral load, it is still a very low viral load as most of these patients have no cultivable virus and are not contagious). | We have rephrased the sentence accordingly (line 61). |

**Introduction:**

| Line 92: according to most experts (e.g: Mina MJ, et al. Rethinking Covid-19 Test Sensitivity - A Strategy for Containment. New England Journal of Medicine. 2020;383(22):e120), RDT for SARS-CoV-2 are central to the fight against the epidemic, not just complementary. | We have rephrased the sentence accordingly and added the mentioned paper as a source (line 118/119). |
| --- | --- |

**Methods:**

| Line 143-147: a comment on the lack of international standard on the viral load categories that should be studied, or at least the viral load thresholds, which impede accurate comparisons should be added. | We have added a respective comment to the methods section (line 173/174) and also included this as one of the study’s limitations in the discussion (line 611/613). |
| --- | --- |

**Results:**

| Line 380-386: Subgroup analysis in symptomatic and asymptomatic patients. Presenting overall results could be misleading as it would suggest that the fact of presenting symptoms or not has a direct influence on the sensitivity, why it could be a confounding factor. Indeed one hypothesis for the observed difference is that the main influencing factor is rather the distribution of viral loads that is different (towards lower loads) in asymptomatic persons than in patients, as well mentioned by authors in the discussion. Therefore it is essential to present results by viral load categories (if available), or at least viral load thresholds, to know if RDT are able to detect significant viral loads (and thus most people who are contagious) also in asymptomatic persons. | Thank you for the suggestion. We fully agree with the comment. Ideally, we would be performing a meta-regression considering presence of symptoms and viral load, however the data allowing to such a subgroup analysis is very scarce. As most data was available for the median Ct-value, we added a statement to the results section, describing how the median Ct-value differed in the symptomatic and asymptomatic subgroup (line 477 to 480). Furthermore, we addressed this aspect in the discussion (line 581 to 583). |
| --- | --- |
| Line 395-406: Subgroup analysis by Ct-values. As mentioned in the General comment, these results are key and should even be the primary outcome measures of such a review. I would thus propose to put this chapter as second chapter in the overall Results section. | For the reasons mentioned above, we did not make it a primary outcome measure. However, we have moved the results of the Ct-value subgroup analysis to be the first paragraph of the subgroup analyses’ results (line 420 to 425). |

**Discussion:**

| Line 449: I would also mention here the 2 results with the Ct thresholds of 25 and 30. | We have presented the Ct-values accordingly (line 568/570). |
| --- | --- |

**Conclusion:**

| As commented above, a viral load corresponding to a Ct of up to 30 is not a high viral load. Indeed most studies suggest that the Ct threshold to differentiate between a culturable form a non-culturable virus is around 25 and than above that threshold most people do not seem to be contagious anymore. | We agree with the reviewer’s comment. However, given that this complex point could be discussed in a separate paper, we refrain from further going into detail on it in the discussion for this paper. We hope the reviewer finds this acceptable. |
| --- | --- |
